# Cholesterol lowering: to live longer, start younger?

**DOI:** 10.18632/aging.102867

**Published:** 2020-02-19

**Authors:** Nelson Wang, Ian Wilcox, Sean Lal

**Affiliations:** 1 Sydney Medical School, University of Sydney, Sydney, Australia; 2 Department of Cardiology, Royal Prince Alfred Hospital, Sydney, Australia; 3 School of Medical Sciences, University of Sydney, Sydney, Australia; 4 The George Institute for Global Health, Sydney, Australia; 5 Central Sydney Cardiology, Sydney, Australia

**Keywords:** LDL cholesterol, cardiovascular risk, primary prevention, secondary prevention, young age

Cardiovascular disease, particularly coronary heart disease and stroke, is a major cause of death globally. Since older age is an independent predictor of increased cardiovascular risk, the global burden of cardiovascular disease increases as populations age. Lowering low density lipoprotein (LDL) cholesterol has become an important strategy for reducing the risk of atherosclerotic cardiovascular disease (ASCVD). Recent evidence from the Cholesterol Treatment Trialist Collaboration [1] has shown that the benefits of this approach extend to patients over 75 years. Though LDL lowering is beneficial across middle to older age, two questions still arise: how early in the disease process should LDL lowering be initiated; and, are the currently recommended LDL targets ambitious enough?

Our study entitled “Intensive LDL cholesterol-lowering treatment beyond current recommendations for the prevention of major vascular events: a systematic review and meta-analysis of randomised trials including 327 037 participants”, published in the January 2020 issue of The Lancet Diabetes and Endocrinology [2], found that lowering LDL cholesterol beyond the current lipid targets further reduces ASCVD risk. Specifically, there was *a* greater relative risk reduction in patients at younger age and lower cardiovascular risk.

The aim of lowering circulating LDL and other apo B–containing lipoproteins is to prevent their deposition within the extracellular matrix of arterial walls and hence minimize the development of atheromatous plaques [3]. In the primary prevention setting, the focus is on the prevention of ASCVD events rather than the development of atherosclerosis per se. Physicians have tended to delay LDL lowering treatment until a patient is considered to have developed a significant risk of ASCVD after many years of exposure to risk factors. For example, the American College of Cardiology/American Heart Association estimated 10-year ASCVD risk for an untreated 50 year old Caucasian male with a systolic/diastolic blood pressure of 140/90 mmHg and LDL cholesterol level of 3.4 mmol/L, is 5.3% [4]. A 60 year old male with the same risk factors has a 10-year ASCVD risk of 11.8%, which increases to 22.6% at age 70. Older age appears to be a marker of the cumulative exposure to LDL cholesterol, along with other traditional cardiovascular risk factors. Delaying treatment until the cardiovascular risk is above a certain threshold will lead to additional years of exposure to this cumulative burden. Initiating statin therapy at say, the age of 50 rather than 60 years old, will prevent an additional 10 mmol/L/year LDL cholesterol exposure, or in other words, provide an additional 10 mmol/L/year of LDL cholesterol reduction.

More complex and older atheromatous plaques only partially regress with LDL lowering therapies, which means delayed treatment will leave older individuals at considerable residual risk of ASCVD. It makes sense to advocate for a greater focus on the lifetime exposure to elevated LDL and the benefit of LDL cholesterol lowering over longer periods of time. A primordial prevention strategy would target the development of atherosclerosis rather than simply preventing its complications. This hypothesis is supported by cohort studies which showed exposure to elevated blood pressure and cholesterol levels during young adulthood is associated with a greater risk of ASCVD later in life, independent of later adult exposures [5].

A primordial prevention approach with earlier LDL cholesterol lowering does not undermine the importance of treating high risk patients, who are still expected to derive the greatest absolute risk reductions. In the secondary prevention setting, there has already been a recent push towards lower LDL targets. Our study and others, have shown that there appears to be no lower limit to the benefits of LDL lowering [2,6,7]. Furthermore, aggressive LDL cholesterol lowering appears to be safe, with no signal towards increased adverse effects even in patients with LDL levels as low as 0.8 mmol/L [7].

How should treating clinicians interpret these recent findings? The goal of therapy in the secondary prevention setting should be to reduce LDL cholesterol as much as tolerated (and affordable), which will often involve combination therapy. The current options of statins, ezetimibe and proprotein convertase subtilisin/kexin type 9 (PCSK9) inhibitors, demonstrate proven efficacy and safety [2]. Newer therapies including bempedoic acid are rapidly emerging and there are ongoing studies investigating a vaccine targeting PCSK9, which would have enormous public health implications [8].

In the primary prevention setting, there should be a push towards treatment at a younger age and therefore a greater focus on primordial prevention. This approach is aimed at preventing the development of atherosclerotic plaques, which across a lifetime, should have large reductions in the risk of a major vascular event. It is obviously important to remember that all treatments have potential adverse effects, financial costs and psychological burden. Discussions about these risks and benefits with our patients remains paramount.
